# Proposed Open-Air Sanatorium for the County of Middlesex

**Published:** 1905-12-09

**Authors:** 


### PROPOSED OPEN-AIR SANATORIUM FOR THE COUNTY OF MIDDLESEX.

The public appeal which has been resolved upon in order to secure sufficient funds for the establishment of an open-air sanatorium for the County of Middlesex has certain special claims upon the attention of the charitably disposed. It is the intention of the promoters that the sanatorium shall have sufficient land attached to it to provide employment for convalescents; that the surroundings and *régime* of the institution shall be such as to encourage the patients to continue the treatment after they return to their own homes; and that the buildings shall be of the simplest and least expensive character. The estimated total outlay on the sanatorium amounts to £300 per bed. We have often enough in these columns insisted on the importance of providing some employment, preferably out of doors, for those patients in sanatoria whose physical condition permits of their undergoing exertion, and also upon the desirability of spending smaller sums on sanatorium buildings than has hitherto been the general custom. We learn, therefore, with considerable satisfaction, that these two principles are to be put in practice in the County of Middlesex Open-air Sanatorium. There can be no question about the present necessity for extended sanatorium accommodation, but this necessity must always be regarded in conjunction with the circumstance that a sanatorium is not so much a place where tuberculous patients may be cured, as a school where consumptives may study and learn the art of self-defence against the attacks of Koch's bacillus. It does considerable credit to the organisers of the Middlesex scheme that they have appreciated this fact.

